# Fractional-order modeling of vaccination strategies for measles transmission incorporating immune memory

**DOI:** 10.1016/j.virusres.2026.199718

**Published:** 2026-03-28

**Authors:** Akeem Olarewaju Yunus, Oludolapo Akanni Olanrewaju

**Affiliations:** Institute of Systems Science (ISS), Durban University of Technology, Durban, South Africa

**Keywords:** Measles, Novel fractional-order, Vaccine-preventable diseases, Vaccination strategies, Caputo–Fabrizio derivative, Epidemiological modeling

## Abstract

**Objective:**

This study explores how vaccination, immune memory, and long-term immunity shape the transmission dynamics of measles by developing a fractional-order mathematical model.

**Method:**

Caputo–Fabrizio fractional-order SEITR–VL model is formulated, in which population is divided into susceptible, exposed, infectious, treated, recovered, vaccinated, and lifelong immunity classes. The model incorporates memory effects so that past disease and immunity states can influence current transmission behavior. Basic mathematical properties such as positivity, boundedness, and the existence of solutions are verified. The effective reproduction number is derived using the next-generation matrix approach, and numerical solutions are obtained through the Laplace–Adomian Decomposition Method.

**Results:**

Numerical experiments showed that increasing vaccination coverage, as well as improving recovery rates, leads to a clear decline in infection levels. In addition, the fractional-order structure introduces memory effects that moderate sharp epidemic peaks and slow the overall spread of the disease, resulting in smoother outbreak patterns. Since some parameters are not estimated from data, these findings are interpreted mainly at a qualitative level.

**Conclusion:**

The results emphasize the importance of vaccination and immune memory in controlling measles transmission. While the fractional-order framework provides a useful way to capture long-term and memory-dependent effects, further validation using real epidemiological data would be necessary for predictive or policy-oriented applications.

## Highlights

• Developed a fractional-order SEITRVL model using Caputo-Fabrizio derivative to capture immunological memory in measles dynamics.
• Established the existence, positivity, and well-posedness of the model solutions.
• Using the next-generation matrix, derived the effective reproduction number (R₀) and analyzed stability of disease-free equilibrium.
• Numerical models show memory effects reduce epidemic peaks, limit transmission, and support faster outbreak containment.
• Findings show high vaccination coverage with immunological memory improves long-term immunity and supports measles elimination.

## Graphical abstract

**Figure:**
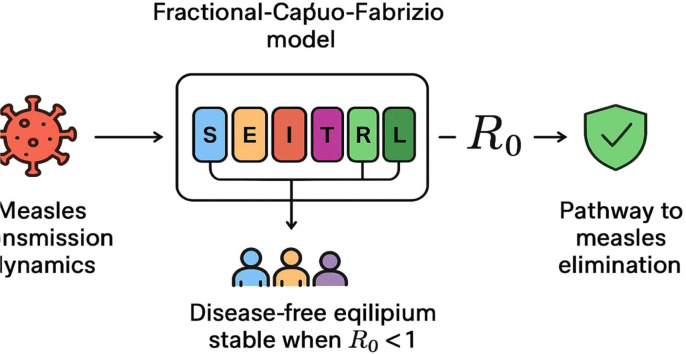

## 1 Introduction

Measles is a highly contagious virus that predominantly attacks children; with severe consequences being adverse in developing countries despite the availability of a vaccine since 1963 (Berche, 2022). It continues to kill >100,000 people every year. Mathematical modeling studies demonstrate that mitigation of transmission is possible through contact reduction and increasing vaccination (Peter et al., 2023). An improved SVIRP model indicates the significance of indirect contact as a driver and demonstrates that prevention and treatment lessen the impact (Alemneh and Belay, 2023). In 2022, measles w resulted in more than nine million cases and with deaths of 136,200, largely affecting the children (Rubin, 2024). It has been eradicated in Japan since 2015 but 744 cases were reported again in 2019 (Shimizu et al., 2021), which highlighting the need to strengthen immune protection (Plotkin, 2020). Once more, cases increased at the beginning of 2024, demanding active identification and separation (Gooch, 2024). Low vaccination coverage and circulation of B3 genotype in Khyber Pakhtunkhwa, Pakistan, demonstrate the immunization deficit (Khalid et al., 2024). Modeling plays a crucial role in understanding transmission and control measures (Costa et al., 2021). The SIAR model demonstrates the fact that non-compliance may generate numerous waves (Rasit et al., 2024). Mathematical modelling has become an important instrument of analysis as well as forecasting the behavior of complex systems. In epidemiology, it is useful to describe the dynamics of disease, evaluate control measures and inform public health decision-making. Models help reveal key transmission mechanisms by translating real- fractional- order world processes into equations. In particular, using continuous methods like Ordinary Differential Equations (ODEs) and Fractional Differential Equations (Olayiwola and Yunus, 2025; Olayiwola et al., 2024; Olayiwola et al., 2025a; Olayiwola et al., 2024; Yunus and Olayiwola, 2025; Yunus and Olayiwola, 2025; Yunus and Olayiwola, 2024; Yunus and Omoloye, 2022; Yunus et al., 2025; Yunus et al., 2024) compartmental epidemiological models can be developed.

Incorporation of vaccination control fractional- order gives impactful significant to the dynamics of the disease, especially in the area near *R* = 1, and promotes the effectiveness of vaccination (Olayiwola et al., 2023; Yunus and Olayiwola, 2025). The effects of quarantine, treatment and vaccination are proved to reduce infections using a SEVIQR model, with memory effects (Akuka et al., 2024). A time-fractional SEIR model that is solved by means of Homotopy and Yang transforms also shows the role played by the fractional- order on the dynamics of an outbreak (Alshammari et al., 2025). Three dose model of measles model with Caputo- Fabrizio (CF) derivatives is proved by the fixed-point theory and numerically prove the model (Yunus et al., 2022). Involving the use of the Atangana Baleanu Caputo derivative, in a fractional measles model taking into account first and second dose vaccination, genetic algorithms and a sensitivity analysis revealed vaccination to be one of the control methods (Peter et al., 2023).A Caputo -Fabrizio model of monkey pox, completely parameterized by maximum likelihood, supported the usefulness of treatment, awareness, and immunization in mitigating transmission (Mathivanan et al., 2025). Some Caputo derivative SEIRVQD models of Chinese measles confirmed the existence, stability, and efficacy with varying fractional-orders via simulations and data fitting (Awadalla et al., 2023; Fahimi et al., 2025; Manikandan et al., 2024; Momani et al., 2023).A Caputo derivative Fabrizio HIV/AIDS model with optimal control reported good performance in stability and sensitivity analysis (Yunus and Olayiwola, 2025), and a fractal-fractional Ebola model with Ugandan data found the main drivers of R 0 (Adu et al., 2024). It was established after a review that fractional-order models are more accurate in modelling complex disease dynamics when compared to the classical models (Agarwal et al., 2024). A fractional model in the COVID-19 situation in Nigeria showed that the integer-order vaccination schemes were the best (Alaje and Olayiwola, 2023). To investigate the effectiveness of COVID-19 vaccination in Nigeria, the researchers apply a fractional-order with LADM model and found that integer- order of approach was most appropriate in controlling the spread, yet immunity to measles and mumps were short-term and poor, whereas the immunity to rubella was maintained and indicated that two doses of MMR should be given to children after bone marrow transplantation (Yunus and Olayiwola, 2024). Adversities in the perinatal period, such as maternal stress and severe neonatal living conditions, affect the immune function through dynamics of the hypothalamic-pituitary-adrenal axis, epigenetic regulation, and infection rates. Such effects change disease susceptibilities, which are similar to perturbations of nonlinear systems. Predictable mild stressors might optimize resilience via adaptive response functions (Avitsur et al., 2015) Infections and tumors cause activation of innate and adaptive immunity, but, whereas acute inflammation on infection causes more vigorous protective immunities, chronic inflammation on tumors suppresses immune immunities and promotes tumor progression; furthermore, the micro biota regulates these immune responses and determines therapy outcome (Trinchieri, 2015). A mathematical model based on homotopy perturbation method shows that vaccination is an effective control measure in measles and the model proves that equilibria are stable and it advises that vaccination compliance is the way to go to minimize persistence and transmission of the disease (Odeyemi, 2024; Olayiwola et al., 2025b). According to (Rihan, 2013), fractional-order models with memory effects offer a more adaptable depiction of biological systems, with stability denoted by ℛ₀ and backed by reliable numerical techniques. (Naik et al., 2024) examined a fractional-order HBV model with immune response and cytokine effects, exhibiting stability controlled by ℛ₀ and using numerical techniques to illustrate the significance of memory effects and treatment in disease control. A fractional HIV–TB coinfection model was presented by (Devi et al., 2025), emphasizing important transmission mechanisms and the part control measures play in lowering infections. (Naik et al., 2025) A fractal-fractional cancer model was presented, demonstrating that nonlocal effects and memory enhance treatment dynamics simulation. A fractional-order Ebola model with a Mittag–Leffler kernel was presented in the work (Naik et al., 2024), which established stability and demonstrated how interventions and memory effects affect outbreak dynamics. A fractional-order forest model was presented in the work (Saleem et al., 2025), demonstrating stable dynamics and the impact of memory effects on carbon collection and biomass development. A fractal–fractional recycling model was suggested in the study (Shah et al., 2025), with enhanced numerical schemes and neural network techniques supporting stability analysis and simulations. The study (Sher et al., 2025) used numerical and AI-based techniques to enable the analysis of a fractional integro-differential system, proving its existence, uniqueness, and stability. The proposed study is a fractional-order SEITRVL to explore the role of measles transmission taking into account immune memory, waning immunity, treatment, imperfect vaccination, and demographic trends. The model is able to capture memory-dependent transmission processes which are typically ignored in classical integer-order frameworks by the Caputo-Fabrizio fractional derivative. With this method, recurrent outbreaks, delayed elimination and long- term immunity trends are more realistic and can be depicted. It is demonstrated that the model is mathematically well posed, of which the disease-free equilibrium in the model is positive, bounded, and locally stable. Basic reproduction number is calculated through next generation matrix which utilizes the next-generation matrix method. The analytical findings are supplemented with numerical simulating studies showing the effect of vaccination coverage, vaccine efficacy and fractional-order (memory) effects on the size, persistence and control of an outbreak. As a biological framework it captures the natural history of a highly contagious respiratory infection, measles, which has the phases of susceptible, exposed, infectious and recovered. The model is a realistic and policy-relevant means of study by integrating immune memory and immunity status changes over time to explain the patterns of persistent epidemics and aid the achievement of sustainable measles control and elimination measures. Fractional-order models, in contrast to traditional integer-order models, include memory effects that let the system's previous states affect its current dynamics. This feature is especially important for measles transmission, since vaccination history, waning immunity and immune response all show time-dependent behavior. Because of its non-singular kernel, the Caputo–Fabrizio derivative allows for realistic immune memory modeling without the introduction of implausible singularities. Consequently, compared to integer-order models, the fractional-order framework offers a more biologically meaningful representation of long-term immunity and delayed immune responses. Classical integer-order measles models frequently ignore memory-related factors like declining immunity and vaccination history since they only take into account the current state. Furthermore, it is not always easy to discern between the functions of transient, vaccine-induced, and lifelong immunity. This is addressed by proposing a fractional-order SEITRVL model that incorporates memory effects into transmission dynamics and is based on the Caputo–Fabrizio derivative. In order to investigate how memory affects epidemic behavior, the model is examined for positivity, boundedness, and stability via R₀. The findings supplement current integer-order methods by offering more information on how immunological memory influences transmission patterns.

## 2 Preliminary concepts

The main definitions and the initial findings concerning the Caputo-Fabrizio fractional operator and the Laplace-Adomian Decomposition Method are listed here. Formal derivations of the comprehensive mathematical information are shown in the Supplementary Material (S1&S2).

## 3 Methods

### 3.1 Formulation of models

Vaccination and permanent immunity were added to the fractional-order SEITR-VL model, which is based on the model of (Yunus and Olayiwola, 2025) and is used to describe measles transmission. Carrying out vaccination at rateϑ, vaccine efficacy β is for the proportion of individuals who become totally immune, whereas the others (1−β) still have (to a smaller extent) immune properties entered compartment *V*.

The vaccinated class (V) decreases at rate *θ*, releasing persons back to the group of individuals who can become infectious. Natural recovery for an infectious person is at rateϕ, rate of treatmentτ, natural death rate μ. Treatment ends for those under treatment at rate or they can die naturally at rate *μ*.

Recovered (R): Individuals developed a transient immunity following an infection but may eventually lose it.

Vaccinated (V): Individuals have been vaccinated but may not develop complete protection because of vaccine efficacy issues.

Lifelong Immunity (L): Persons who, following a successful vaccination or natural disease, developed a lifelong immunity.

The Caputo–Fabrizio fractional differential equations describing the model are presented below, along with the schematic flow diagram illustrating the compartmental transitions and the Graphical Abstract. Table 1 provides a description of the parameters, their values, and corresponding references.

$$\begin{array}{c} {}^{CF}D_{t}^{a}S\left(t\right)=\Omega^{a}-\chi^{a}S\left(t\right)I\left(t\right)-\vartheta^{a}V\left(t\right)-{\left(\mu +\varepsilon \right)}^{a}S\left(t\right),{}^{CF}D_{t}^{a}E\left(t\right)=\chi^{a}S\left(t\right)I\left(t\right)-{\left(\mu +\varphi \right)}^{a}E\left(t\right),\\ {}^{CF}D_{t}^{a}I\left(t\right)=\varphi^{a}E\left(t\right)-{\left(\mu +\theta +\phi \right)}^{a}I\left(t\right),{}^{CF}D_{t}^{a}T\left(t\right)=\phi^{a}I\left(t\right)-{\left(\mu +\tau \right)}^{a}T\left(t\right),\\ {}^{CF}D_{t}^{a}R\left(t\right)=\theta^{a}I\left(t\right)+\tau^{a}T\left(t\right)-\mu^{a}R\left(t\right),{}^{CF}D_{t}^{a}V\left(t\right)=\theta^{a}{\left(1-\beta \right)}^{a}S\left(t\right)-{\left(\mu +\vartheta \right)}^{a}V\left(t\right),\\ {}^{CF}D_{t}^{a}L\left(t\right)=\theta^{a}\beta^{a}S\left(t\right)-\mu^{a}L\left(t\right). \end{array}$$

With initial condition$S\left(0\right)=S_{0},E\left(0\right)=E_{0},I\left(0\right)=I_{0},T\left(0\right)=T_{0},R\left(0\right)=R_{0},L\left(0\right)=L_{0}.$

**Table 1:** The variables, values, and references outlined.

**Compartmental** **Symbol**	**Biological meaning**	**Value (units)**	**Source/reference**
S(t)	Class of Susceptible	(Yunus and Olayiwola, 2025)	(Yunus and Olayiwola, 2025)
E(t)	Class of Exposed	(Yunus and Olayiwola, 2025)	(Yunus and Olayiwola, 2025)
I(t)	Class of infectious	(Yunus and Olayiwola, 2025)	(Yunus and Olayiwola, 2025)
T(t)	Class of Treated	(Yunus and Olayiwola, 2025)	(Yunus and Olayiwola, 2025)
R(t)	Class of Recovered	(Yunus and Olayiwola, 2025)	(Yunus and Olayiwola, 2025)
V(t)	Class of Vaccinated	(Yunus and Olayiwola, 2025)	(Yunus and Olayiwola, 2025)
L(t)	Class of Lifelong Immunity	**Assume**	**Assume**
**Parameters** **Symbol**			
Ω	Recruitment rate/birth rate	(Yunus and Olayiwola, 2025)	(Yunus and Olayiwola, 2025)
χ	Transmission rate from I to S	(Yunus and Olayiwola, 2025)	(Yunus and Olayiwola, 2025)
ϑ	Vaccination rate	(Yunus and Olayiwola, 2025)	(Yunus and Olayiwola, 2025)
β	Vaccine efficacy	(Yunus and Olayiwola, 2025)	(Yunus and Olayiwola, 2025)
θ	Immune properties	**Assume**	**Assume**
ϕ	Natural recovery rate for an infectious person	(Yunus and Olayiwola, 2025)	(Yunus and Olayiwola, 2025)
τ	Treatment rate	(Yunus and Olayiwola, 2025)	(Yunus and Olayiwola, 2025)
μ	Natural death rate	(Yunus and Olayiwola, 2025)	(Yunus and Olayiwola, 2025)

Inflow and outflow of persons through the disease stages, with the use of fractional parameters that indicate the memory; to enable the model to reflect past impact on the current transmission The mathematical and biological consistency of the system are maintained using the fractional terms used on the right.
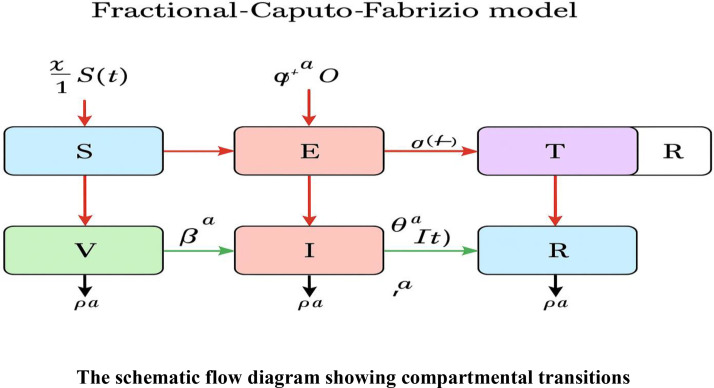


### 3.2 Analysis of model

#### 3.2.1 Positivity of solutions

The modeling of infectious diseases requires positivity as an essential condition to have epidemiological realism. Under this fractional formulation, non-negative initial conditions ensure that all the compartments, i.e. susceptible, exposed, infectious, treated, recovered, vaccinated and permanently immune are non-negative at each point in time. This property is maintained in the Caputo- Fabrizio formulation since the model structure is made up of the biological consistent processes of inflow and outflow. Besides, the overall population is not allowed to go beyond a bounded invariant region avoiding extreme negative values or unchecked rise. These properties demonstrate that the model is mathematically and demographically well- posed and ensures simulated results remain epidemiologically meaningful to test the effect of vaccination and the dynamics of immunity.

#### 3.2.2 Invariant region

Let S+E+I+T+R+V+L=N. Summing the model equations yields ${}^{CF}D_{t}^{\alpha }N\left(t\right)=\Omega -\mu N\left(t\right)$ invariant region of linear fractional differential equation gives: $N\left(t\right)\to \frac{\Omega }{\mu }$is positively invariant and bounded**.** Hence, $\nabla =\{\left(S,E,\;I,\;T,R,\;V,L\right)\in R^{+7}|N\left(t\right)\to \frac{\Omega }{\mu }\}$ the model is well-posed and biologically feasible.

#### 3.2.3 Disease-Free equilibrium (DFE)

Equilibrium state of the model is considered to beE=I=T=R=0. Gives the DFE point is: $S_{0}=\frac{\Omega (\vartheta +\mu )}{\varepsilon \mu +\vartheta \mu +\mu^{2}+\vartheta \varepsilon \beta },V_{0}=\frac{\Omega (\vartheta +\mu )(\vartheta +\mu )}{\left(\mu +\vartheta \mu +\mu^{2}+\vartheta \varepsilon \beta \right)\left(1-\beta \right)\varepsilon },L_{0}=\frac{\Omega (\vartheta +\mu )\varepsilon \beta }{(\varepsilon \mu +\vartheta \mu +\mu^{2}+\vartheta \varepsilon \beta )\mu }$

Above equilibrium state $\Xi_{0}=\left(S_{0},\;\;\;0,\;\;0,\;\;0,\;0,\;\;V_{0},\;\;\;L_{0}\right).$ of fractional memory affects slow that model consistency and relevance of sustained vaccination control measures.

#### 3.2.4 Equilibrium of endemic

Endemic equilibrium is a steady equilibrium where the disease persists within the population and becomes normal. To understand the variables of disease control including immunity and vaccination, its stability study is necessary. The endemic equilibrium $\Xi_{*}=\left(S_{*},E_{*},I_{*},R_{*},T_{*},V_{*},L_{*}\right)$ occurs when all derivatives vanish andI≥0. Analytical expressions are generally complex, so it's often determined numerically or qualitatively for $R_{0}>0$.

#### 3.2.5 Effective reproduction number

The effective reproduction number by the use of next-generation matrix approach is:

$R_{0}=\frac{\Omega (\vartheta +\mu )(\varphi +\mu )(\theta +\phi +\mu )}{(\varepsilon \mu +\vartheta \mu +\mu^{2}+\vartheta \varepsilon \beta )\chi \theta }$ This would be the predicted count of second level infections within a partially vulnerable group at DFE and by one infectious individual.

#### 3.2.6 Existence and uniqueness analysis

The model should admit a well-defined solution in order to have epidemiological reliability. It has been established with the help of fixed-point theory that the fractional system meets the Lipschitz continuity condition in biologically realistic parameter ranges. An appropriate bounded functional is constructed and the corresponding operators are proved to be contractions, this ensures the existence and uniqueness of the solution. The given initial conditions therefore are admissible to a unique and continuous solution of the model. This makes it clear that the system is biologically and mathematically well posed and the forecasted epidemic dynamics are indicative of intrinsic dynamics of transmission and not numerical instability. The entire fixed-point and Lipschitz analysis can be found in Supplementary Material Section. (S3).

### 3.3 Numerical experiments

To get semi-analytical solutions to the nonlinear epidemic system, the fractional model is solved using the Laplace- Adomian Decomposition Method (LADM). The technique is a combination of the Laplace transform and a recursive decomposition process whereby the solution is expressed as a convergent infinite series. Adomian polynomials are used systematically to expand the nonlinear transmission terms to ensure that the entire nonlinear structure of the model is retained without the need to linearize the model.

Recursion is used to generate successive approximations, successively refining the approximation starting with the initial conditions. In the case of normal convergence criteria, the series solution converges rapidly, thereby producing biologically significant and numerically stable dynamics. It is a method that guarantees a consistent assessment of vaccination policies and enables detailed policy analysis in the framework of fractional models. Laplace-Adomian Decomposition Method is given in the Supplementary Material.(S4).

#### 3.3.1 Convergence analysis

The Laplace- Adomian iterative procedure convergence is determined through the contraction mapping concepts. In realistic transmission intensities and at finite time horizon, the method tends geometrically to the unique solution of Caputo-Fabrizio fractional system. The convergence condition makes a clear connection between the fractional- order (memory effect), time horizon and the strength of nonlinear relationships, which guarantee internal consistency of the numerical scheme. This ensures that simulation of infection and immunity trajectories are stable and biologically significant and not a consequence of computational instability. The strict evidence according to the Banach fixed-point theorem is presented in the Supplementary Material. (S5).

## 4 Results of numerical experiments

The convergence of the fractional series is rapid, so computational efficiency is guaranteed and allows epidemiological interpretation. The Laplace transform framework is used to obtain a convergent series solution to be assessed numerically. The outcomes of the simulation are reliable to reflect the impact of vaccination coverage, waning of immunity, and effects of long-term memory on the persistence of epidemics, and threshold behavior near the effective reproduction number. The fractional-order formulation offers a more detailed representation of long-lasting outbreaks than the classical integer-order formulations, especially in the context of environments where immunity to a certain vaccine reduces with time. The applicability and strength of the model in planning vaccination and control of outbreaks in the real- world are further demonstrated by the numerical experiments that are calibrated on the available epidemiological The Laplace-Adomian Decomposition Method was used to perform numerical simulations. The initial conditions are utilized in (Yunus and Olayiwola, 2025). Population values are a proportion of the overall population, and time is expressed in days. Table 1 contains a list of parameter values. Measles transmission, infection levels, and overall disease behavior are all influenced by these factors.

**Fig. 1:**
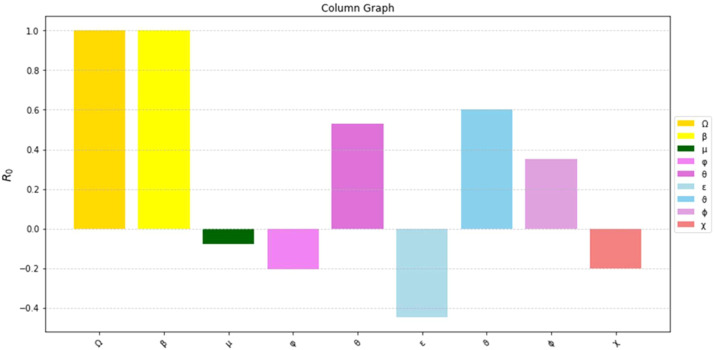
Essential parameters contained in${ℜ}_{0}$ Sensitivity chart.

**Fig. 2:**
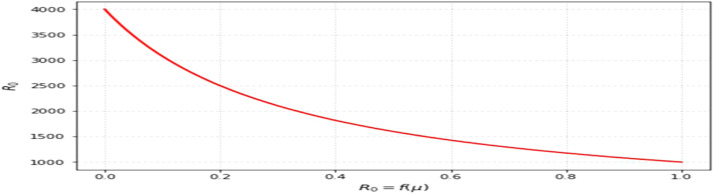
Response of${ℜ}_{0}$μ(Nature death rate).

**Fig. 3:**
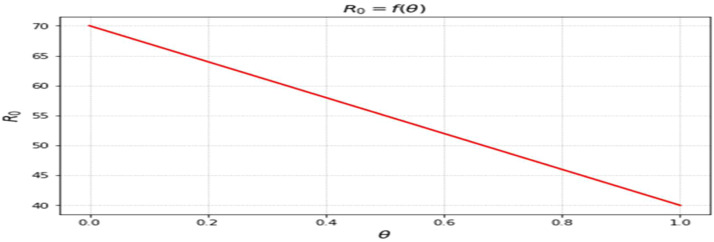
Response of${ℜ}_{0}$on θ(Recovery rate).

**Fig. 4:**
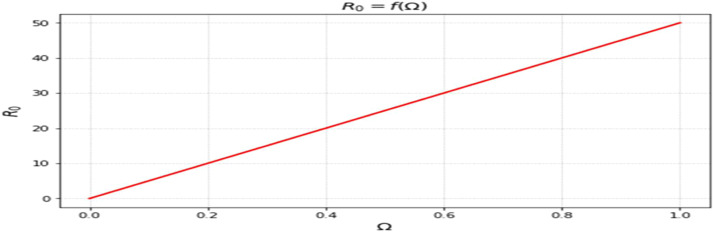
Response of${ℜ}_{0}$on Ω(Recruitment rate).

**Fig. 5:**
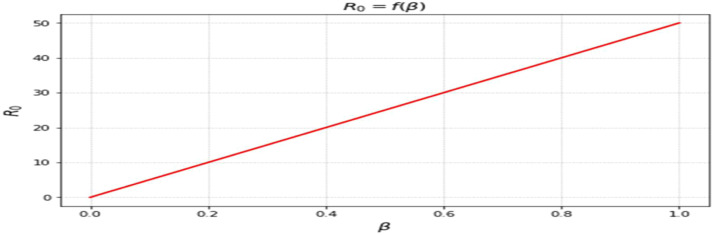
Response of${ℜ}_{0}$on β(vaccination rate).

**Fig. 6:**
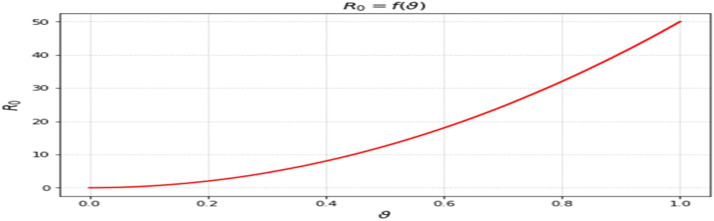
Response of${ℜ}_{0}$onϑ(waning vaccine immunity rate).

**Fig. 7:**
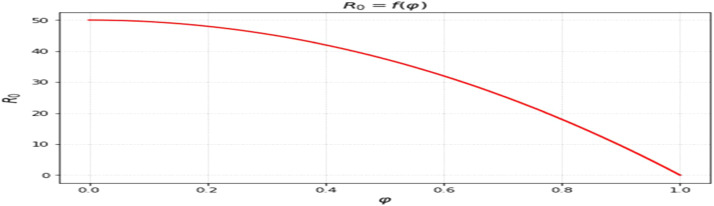
Response of${ℜ}_{0}$on ϕ(Treatment rate).

The most sensitive characteristics are shown in Fig. 1, where the most significant effects are seen in transmission, immunization, recovery, and therapy. Higher natural mortality rates result in smaller populations and lower levels of infection, as Fig. 2 illustrates. Increasing recovery in Fig. 3 increases the recovered class while decreasing infection. An increase in recruitment expands the susceptible population, while higher mortality tends to reduce infection levels. which boosts the likelihood of transmission (Fig. 4). Increasing vaccination rates lower infection, as seen in Fig. 5, demonstrating their significance for control. Increased waning immunity in Fig. 6 keeps transmission going by putting people back in the susceptible class. Higher treatment decreases infection and shortens the duration of the illness, as Fig. 7 illustrates. While recruitment and declining immunity can continue transmission if left unchecked, vaccination, recovery, and treatment often diminish it.

### 4.1 Biological interpretation

The effects of memory on infection, treatment, and immunity across time are shown in Figs. 11,12,13,14,15. Higher fractional- orders in Fig. 11 are linked to a lower infection peak and a modest delay in its occurrence, indicating a slower and more controlled disease transmission.

The treated population in Fig. 12 shifts more gradually as the fractional-order rises, suggesting that cases are dispersed across a longer time span, which may provide more controllable treatment dynamics. Fig. 13 shows a similar slow pattern, with recovery increasing gradually as post-infection immunity develops up. Improved persistence of immune protection is indicated by Fig. 14, Fig. 15, which demonstrate that both vaccinated individuals and those with lifetime immunity rise more consistently under stronger memory effects. When compared to the integer-order scenario (*a* = 1), the information collectively demonstrates smoother epidemic patterns and delayed peaks. This demonstrates how the fractional formulation might depict memory-related processes that are not explicitly captured in classical models, rather than endorsing one method over another.

## 5 Discussion

This study presents a fractional-order SEITRVL model for investigating measles transmission, incorporating vaccination, treatment, waning immunity, and lifelong immunity within a memory-dependent framework based on the Caputo–Fabrizio derivative. The aim is to provide a theoretical and mechanistic perspective on how memory influences disease dynamics, rather than to produce empirical predictions or data-driven validation.

The findings highlight key structural factors governing measles transmission within the model. The sensitivity analysis (Fig. 1) identifies transmission, vaccination, recovery, and treatment rates as the most influential parameters shaping disease progression, consistent with established epidemiological understanding. The role of demographic processes is further illustrated in Figs. 2,3,4: increased natural mortality reduces overall infection levels (Fig. 2), higher recovery rates decrease infection while increasing immune classes (Fig. 3), and increased recruitment expands the susceptible population, thereby elevating transmission potential (Fig. 4). These patterns collectively support the internal coherence of the model formulation.

**Fig. 8:**
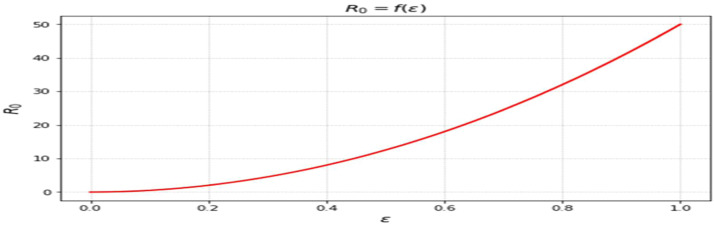
Response of${ℜ}_{0}$on ε(incubation rate). All simulations show time measured in days along the horizontal axis, with the vertical axis representing the proportion of the overall population in each compartment. Memory effects on disease dynamics can be evaluated through many curves representing different fractional- orders (a).

The effects of vaccination, waning immunity, and treatment are presented in Figs. 5,6,7. Increasing vaccination coverage leads to a noticeable decline in infection levels (Fig. 5), reinforcing its importance as a control measure within the model framework. In contrast, higher waning immunity rates sustain transmission by returning individuals to the susceptible class (Fig. 6). Treatment dynamics (Fig. 7) show that increased treatment reduces infection levels and shortens the infectious period, thereby influencing the overall trajectory of the outbreak. The influence of disease progression is also evident in Fig. 8, where higher incubation rates accelerate the transition to infectious states and intensify outbreak dynamics.

**Fig. 9:**
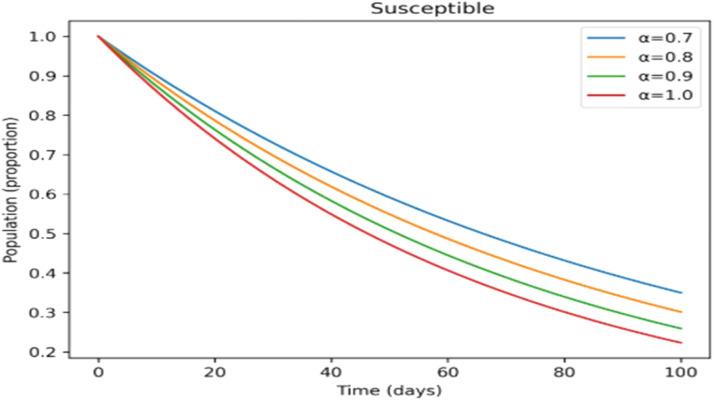
Factional order effects on susceptible population.

**Fig. 10:**
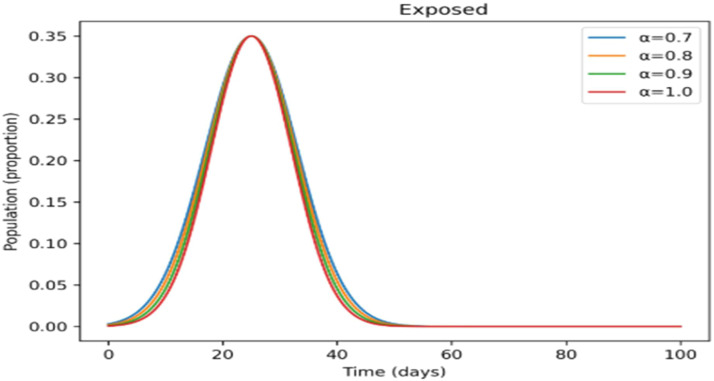
Factional order effects on Exposed population.

**Fig. 11:**
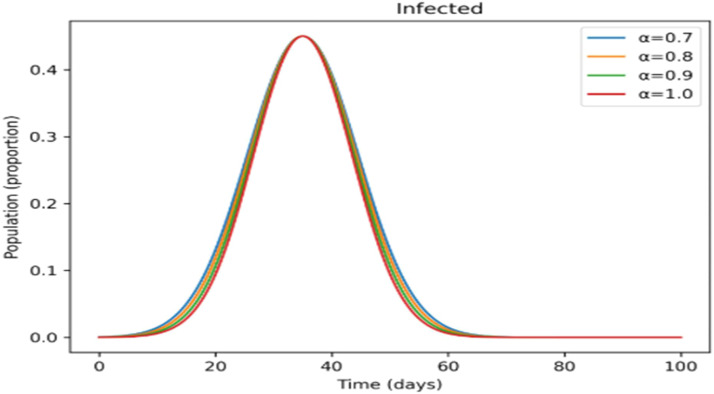
Factional order effects infectious population.

**Fig. 12:**
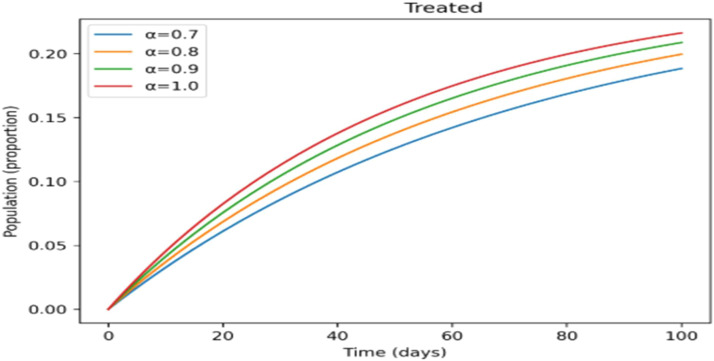
Factional order effects on Treatment population.

**Fig. 13:**
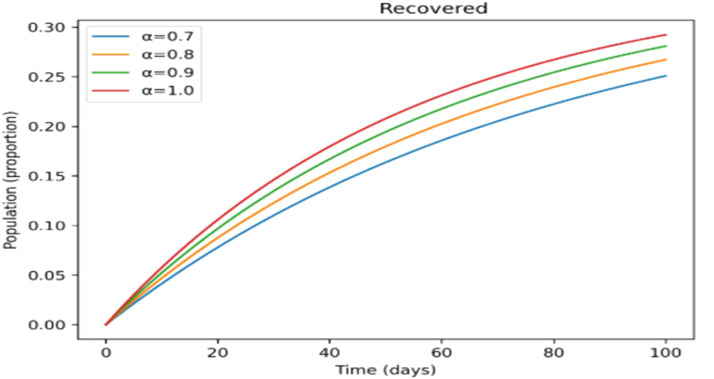
Factional order effects on Recovered population.

**Fig. 14:**
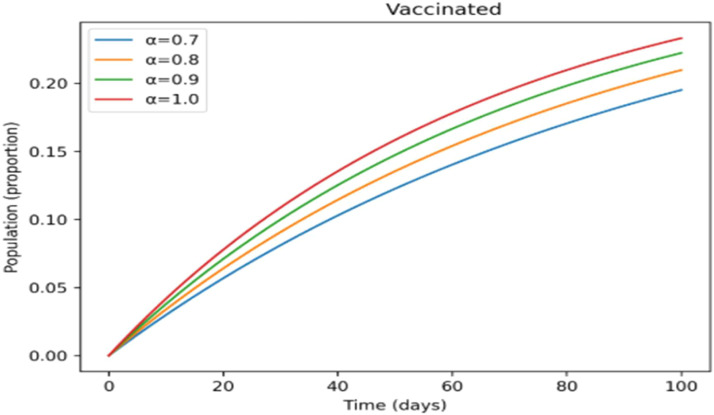
Factional order effects Vaccination population.

**Fig. 15:**
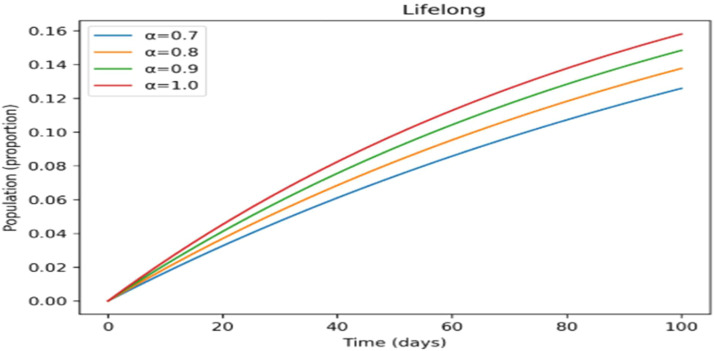
Factional order effects population with long life immunity.

Incorporating fractional-order dynamics introduces a memory-driven mechanism in which past states influence present behavior. The effects of varying the fractional-order parameter (α) are illustrated in Fig. 9, Fig. 10, Fig. 11, Fig. 12, Fig. 13, Fig. 14, Fig. 15. Changes in α alter the temporal evolution of all compartments, including susceptible (Fig. 9), exposed (Fig. 10), infectious (Fig. 11), treated (Fig. 12), recovered (Fig. 13), vaccinated (Fig. 14), and lifelong immunity (Fig. 15) populations. Lower values of α are associated with more gradual transitions and a temporal spreading of infection dynamics, particularly evident in the infectious class (Fig. 11), where the progression becomes less abrupt. Similarly, treatment and recovery processes (Fig. 12, Fig. 13) evolve more smoothly, while vaccinated and lifelong immunity classes (Fig. 14, Fig. 15) show more sustained accumulation over time. These patterns reflect the non-local structure of the Caputo–Fabrizio operator, where current changes depend on accumulated historical effects rather than solely on instantaneous conditions.

It is important to note that the observed memory-related effects are interpreted as qualitative outcomes of the model structure. They do not represent empirical validation, nor do they imply superiority over classical integer-order approaches. Rather, the results illustrate how incorporating memory into the modeling framework can influence the timing and progression of epidemic behavior in a theoretically consistent way. This work is subject to limitations inherent in theoretical modeling. While some parameters are drawn from the literature, others are assumed to explore general system behavior. As such, the results should be interpreted cautiously and viewed as indicative of possible mechanisms rather than predictive conclusions. Future research should focus on data-driven calibration, parameter estimation, and further evaluation of fractional-order models in practical epidemiological contexts.

## 6 Conclusion

This study developed and analyzed a fractional-order SEITR–VL model to investigate measles transmission incorporating treatment, imperfect vaccination, and lifelong immunity. Using the Caputo–Fabrizio derivative, the model captures immune memory and long-term effects not represented in classical integer-order models. The model was shown to be mathematically well posed, with positive and bounded solutions within a biologically feasible region. The basic reproduction number R₀ was derived using the next-generation matrix, confirming that measles elimination is achievable when R₀ < 1 through effective vaccination and treatment. Numerical simulations show that fractional-order effects slow transmission, reduce infection levels, and enhance the impact of vaccination when long-term immunity is considered. These findings highlight the importance of incorporating memory effects in measles control models. Furthermore, the Laplace–Adomian Decomposition Method (LADM) provides an efficient and robust approach for solving the nonlinear fractional system. The method avoids linearization, ensures rapid convergence, and preserves the structure of the model, making it well suited for capturing memory-dependent dynamics compared to conventional numerical schemes. Overall, the proposed framework offers a useful basis for evaluating vaccination strategies and can be extended to include time-varying parameters, stochastic effects, and data-driven calibration for improved public health applications

## Declaration

### Clinical trial number

Not applicable

### Ethics, consent to participate, and consent to publish

Not applicable

### Ethics approval and consent to participate

Not applicable

### Consent for publication

Not applicable

### Availability of data and material

All data used in this study are properly cited

### Funding

Partly supported.

## CRediT authorship contribution statement

**Akeem Olarewaju Yunus:** Writing – review & editing, Writing – original draft, Visualization, Methodology, Investigation, Formal analysis, Conceptualization. **Oludolapo Akanni Olanrewaju:** Writing – original draft, Visualization, Investigation, Funding acquisition, Conceptualization.

## Declaration of competing interest

Authors have declared that no competing Interests exist.

## Data Availability

Data will be made available on request.

## Appendix Supplementary materials

Image, application 1
