# Supplementary material for: Fractional-order modeling of vaccination strategies for measles transmission incorporating immune memory
Source: Virus Res. 2026 Mar 28;367:199718. doi: 10.1016/j.virusres.2026.199718 (PMC13091136; doi:10.1016/j.virusres.2026.199718)
Supplement: Supplementary file 1 [file mmc1.docx]

**Supplementary Material.**

The mathematical foundations of the Caputo–Fabrizio fractional operator, the detailed Laplace–Adomian decomposition procedure, and the complete convergence proof are provided in the Supplementary Material.

**S1. Preliminaries: Caputo–Fabrizio Fractional Operator**

**Definition S1. 1: [42]** Let $x\in G^{'}\left[0,J\right], J>0, b\in\left(0,1\right),$CF gives

: $^{CF}I_{t}^{a}\left(x\left(t\right)\right)=\frac{F\left(b\right)}{1-b}\int x'\left(y\right)e^{-\left[q\frac{t-b}{1-b}\right]}dy$ $\forall F\left(b\right)$ Normalization function ,$\Rightarrow F\left(0\right)=F\left(b\right)=1$.

**Definition S1 .2: [42]** CF with$q\in\left(0,1\right),$ defined as $^{CF}I_{t}^{q}\left[x\left(t\right)\right]=\frac{2\left(1-b\right)}{\left(1-b\right)F\left(b\right)}x\left(t\right)+\frac{2b}{\left(2-b\right)F\left(b\right)}\int_{0}^{t} y\left(x\right)dy,$ $t\geq0$

**Definition S1.3: [42]** C-F of order $b\in\left(0,1\right),$The Laplace Transform defined as $L\left[^{CF}I_{t}^{b}x\left(t\right)\right]=\frac{wL\left[x\left(t\right)\right]-f\left(0\right)}{w+b\left(1-w\right)}$, $w\geq0$

**Definition S1.4: [42]** $D_{0},D_{1,....}D_{n}$,is the Adomian polynomials consists $\chi(t)$which is the expressed as$\chi(t)=\chi_{0}+\chi_{1}+\chi_{2}+\cdots\chi_{n}$ is given as:

$$\zeta_{n}=\frac{1}{n}\frac{d^{n}}{d\lambda^{n}}\left[\chi(t)\sum_{j=0}^{n} \chi_{j}\lambda^{j}\right]_{\lambda=0}$$

**S2: Laplace-Adomian Decomposition Method Flow Chat [42]**

Look at differential equation of fractional-order that follows

;$_{t}^{CF}I^{\alpha}y_{i}(t)=M_{i}(\chi_{1},\chi_{2},\chi_{3},\cdots\chi_{i})+N_{i}(\chi_{1},\chi_{2},\chi_{3},\cdots\chi_{i}).$ (1)

Subject to

$\chi_{i}^{k_{i}}(0)=c_{k}^{i}$, $\forall i=1,2,3...m,$ and$n_{i-1}\leq\alpha\leq n_{i}$.

Where$M_{i}$ linear $N_{i}$ nonlinear differential operators

$_{t}^{CF}D^{\alpha}y_{i}(t)$ CF operator of $i$ number $\chi(t)$,

We begin (1) using LADM.

$L\left[ _{t}^{CF}D^{\alpha}\chi_{i}(t) \right]=L\left[ M_{i}(\chi_{1},\chi_{2},\chi_{3},\cdots\chi_{i})+N_{i}(\chi_{1},\chi_{2},\chi_{3},\cdots\chi_{i}) \right]$. (2)

Using definition 3 on (2) results in:

$\frac{wL\left[\chi_{i}\left(t\right)\right]-\chi_{i}\left(0\right)}{w+\alpha\left(1-w\right)}=L\left[ M_{i}(\chi_{1},\chi_{2},\chi_{3},\cdots\chi_{i})+N_{i}(\chi_{1},\chi_{2},\chi_{3},\cdots\chi_{i}) \right]$. (3)

The unknown function is broken down as follows using the Adomian decomposition method:

$\chi_{i}(t)=\sum_{j=0}^{\infty} \chi_{ij}(t),$ $i=1,2,3\cdots,m$, (4)

Nonlinear terms as:

$N_{i}(\chi_{1},...\chi_{i})=\sum_{j=0}^{\infty} B_{ij}(t),$ $i=1,2,...m$. (5)

Additionally, definition 4 defines $D_{ij}$ as the Adomian polynomial. Afterwards, evaluating (3) using (4) and (5) results in:

:

$L\left[\sum_{j=0}^{\infty} \chi_{ij}(t)\right] =\frac{\chi_{i}^{k_{i}}(0)}{w}+\frac{s+\alpha\left(1-w\right)}{w}\left(L \left[L_{i}\left(\sum_{j=0}^{\infty} \chi_{1j}(t),...,\sum_{j=0}^{\infty} \chi_{mj}(t)\right)\right]+L\left[\sum_{j=0}^{\infty} B_{ij}(t)\right]\right)$ (6)

$\chi_{i(j+1)}(t)=L^{-1}\left(\frac{w_{i}^{k_{i}}(0)}{w}\right)+L^{-1}\left(\frac{w+\alpha\left(1-s\right)}{w}L\left[ L_{i}\left(\sum_{j=0}^{\infty} w_{1j}(t),...,\sum_{j=0}^{\infty} w_{mj}(t)\right)\right]+L\left[\sum_{j=0}^{\infty} B_{ij}(t)\right]\right)$.

It reduces to the necessary recurrence relation provided by

$\chi_{i(j+1)}(t)=c_{k}^{i}+L^{-1}\left(\frac{w+\alpha\left(1-w\right)}{w}L\left[ L_{i}\left(\sum_{j=0}^{\infty} \chi_{1j}(t),...,\sum_{j=0}^{\infty} \chi_{mj}(t)\right)\right]+L\left[\sum_{j=0}^{\infty} B_{ij}(t)\right]\right)$ (7)

**S3. Existence and Uniqueness Proof**

**Existence and uniqueness analysis**

$$\begin{matrix} {}^{CF}D_{t}^{a}S(t)=\Omega^{a}-\chi^{a}S(t)I(t)-\vartheta^{a}V(t)-\left(\mu+\varepsilon\right)^{a}S\left(t\right), \\ {}^{CF}D_{t}^{a}E(t)=\chi^{a}S(t)I(t)-\left(\mu+\varphi\right)^{a}E\left(t\right), \\ {}^{CF}D_{t}^{a}I(t)=\varphi^{a}E(t)-\left(\mu+\theta+\phi\right)^{a}I\left(t\right), \\ {}^{CF}D_{t}^{a}T(t)=\phi^{a}I(t)-\left(\mu+\tau\right)^{a}T\left(t\right), (8) \\ {}^{CF}D_{t}^{a}R(t)=\theta^{a}I(t)+\tau^{a}T\left(t\right)-\mu^{a}R(t), \\ {}^{CF}D_{t}^{a}V(t)=\theta^{a}(1-\beta)^{a}S(t)-(\mu+\vartheta)^{a}V(t), \\ {}^{CF}D_{t}^{a}L(t)=\theta^{a}\beta^{a}S(t)-\mu^{a}L(t). \end{matrix}$$

It is essential to ascertain whether a mathematical model has a solution. By creating the following functional for model , the fixed-point theory technique and Lipchitz condition can be used to address this:

$\left.\begin{matrix} _{0}^{CF}D_{t}^{\alpha}S\left(t\right)=U_{1}\left(t,S\left(t\right)\right)_{0}^{CF}, D_{t}^{\alpha}E_{1}\left(t\right)=U_{2}\left(t,E\left(t\right)\right), _{0}^{CF}D_{t}^{\alpha}I\left(t\right)=U_{3}\left(t,I\left(t\right)\right), \\ _{0}^{CF}D_{t}^{\alpha}T\left(t\right)=U_{4}\left(t,T\left(t\right)\right), _{0}^{CF}D_{t}^{\alpha}R\left(t\right)=U_{5}\left(t,R\left(t\right)\right), _{0}^{CF}D_{t}^{\alpha}V\left(t\right)=U_{6}\left(t,V\left(t\right)\right), \\ _{0}^{CF}D_{t}^{\alpha}L\left(t\right)=U_{7}\left(t,L\left(t\right)\right).. \end{matrix}\right\}$ (9)

By definition **S1.2** on (9)

$\begin{matrix} S\left(t\right)-S\left(0\right)=\frac{2\left(1-a\right)}{\left(2-a\right)F\left(a\right)}U_{1}\left(t,S\left(t\right)\right)+\frac{2a}{\left(2-a\right)F\left(a\right)}\int_{0}^{t} U_{1}\left(a,S\right)da, \\ E\left(t\right)-E\left(0\right)=\frac{2\left(1-a\right)}{\left(2-a\right)F\left(a\right)}U_{2}\left(t,E\left(t\right)\right)+\frac{2a}{\left(2-a\right)F\left(a\right)}\int_{0}^{t} U_{2}\left(a,E\right)da, \\ I\left(t\right)-I\left(0\right)=\frac{2\left(1-a\right)}{\left(2-a\right)F\left(a\right)}U_{3}\left(t,I\left(t\right)\right)+\frac{2a}{\left(2-a\right)F\left(a\right)}\int_{0}^{t} U_{3}\left(a,I\right)da, \\ T\left(t\right)-T\left(0\right)=\frac{2\left(1-a\right)}{\left(2-a\right)F\left(a\right)}U_{4}\left(t,T\left(t\right)\right)+\frac{2a}{\left(2-a\right)F\left(a\right)}\int_{0}^{t} U_{4}\left(a,T\right)da, \\ R\left(t\right)-R\left(0\right)=\frac{2\left(1-a\right)}{\left(2-a\right)F\left(a\right)}U_{5}\left(t,R\left(t\right)\right)+\frac{2a}{\left(2-a\right)F\left(a\right)}\int_{0}^{t} U_{5}\left(a,R\right)da, \\ V\left(t\right)-V\left(0\right)=\frac{2\left(1-a\right)}{\left(2-a\right)F\left(a\right)}U_{6}\left(t,V\left(t\right)\right)+\frac{2a}{\left(2-a\right)F\left(a\right)}\int_{0}^{t} U_{6}\left(a,V\right)da, \\ L\left(t\right)-L\left(0\right)=\frac{2\left(1-a\right)}{\left(2-a\right)F\left(a\right)}U_{7}\left(t,L\left(t\right)\right)+\frac{2a}{\left(2-a\right)F\left(a\right)}\int_{0}^{t} U_{7}\left(a,L\right)da. \end{matrix}$ (10)

**Theorem S3. 1**

The Lipchitz criterion of contraction is satisfied by kernel $U_{1}$ this inequality hold;

$$0\leq\left(\chi^{a}\left\|I\right\|+\mu^{a}+\varepsilon^{\alpha}\right)<1$$

**Proof**

$$\left\|U_{1}\left(t,S\right)-U_{1}\left(t,S_{1}\right)\right\|=\left\|-\chi^{a}I\left[S\left(t\right)-S_{1}\left(t\right)\right]-\left[\mu^{a}+\varepsilon^{a}\right]\left[S\left(t\right)-S_{1}\left(t\right)\right]\right\|$$

$$\begin{matrix} \leq\left\|I\right\| \left\|S\left(t\right)-S_{1}\left(t\right)\right\|+\left\|\mu^{a}+\varepsilon^{a}\right\|\left\|S\left(t\right)-S_{1}\left(t\right)\right\|\leq\left\|S\left(t\right)-S_{1}\left(t\right)\right\| \left\|S\left(t\right)-S_{1}\left(t\right)\right\|\left\|\chi^{a}\right\|+\left.\left(\mu^{^{a}}+\varphi^{a}\right)\right\|. \\ (11) \end{matrix}$$

Let$C_{1}=\chi^{a}\left\|I\right\|+\left.\left(\mu^{a}+\varepsilon^{a}\right)\right\|$, and$||I||\leq\delta_{1}$, is bounded such that

$\left\|U_{1}\left(t,S\right)-U_{1}\left(t,S_{1}\right)\right\|\leq C_{1}\left\|S\left(t\right)-S_{1}\left(t\right)\right\|$ Then Lipchitz condition is satisfied for$C_{1}$, $0\leq\beta^{a}\left\|I\right\|+\mu^{a}+\varphi^{a}<1$and $C_{1}$contracts.

moreso Lipchitz condition of other functional are given by

$\left\|U_{2}\left(t,E\right)-U_{2}\left(t,E\right)\right\|\leq C_{2}\left\|E\left(t\right)-E\left(t\right)\right\|$, $\left\|U_{3}\left(t,I\right)-U_{3}\left(t,I\right)\right\|\leq C_{3}\left\|I\left(t\right)-I\left(t\right)\right\|$

$\left\|U_{4}\left(t,T\right)-H_{4}\left(t,T\right)\right\|\leq C_{4}\left\|T\left(t\right)-T\left(t\right)\right\|$, $\left\|U_{5}\left(t,R\right)-U_{5}\left(t,R\right)\right\|\leq U_{5}\left\|R\left(t\right)-R\left(t\right)\right\|$

$\left\|U_{6}\left(t,V\right)-U_{6}\left(t,V\right)\right\|\leq C_{6}\left\|V\left(t\right)-V\left(t\right)\right\|$, $\left\|U_{7}\left(t,L\right)-U_{7}\left(t,L\right)\right\|\leq C_{7}\left\|L\left(t\right)-IL\left(t\right)\right\|$. (12)

And a contraction occurs for each

$C_{2}=0\leq\mu^{a}+\varphi^{a}<1$, $C_{3}=0\leq\mu^{a}+\theta^{a}+\phi^{a}<1$, $C_{4}=0\leq\mu^{a}+\tau^{a}<1$,$C_{5}=0\leq\mu^{a}+\vartheta^{a}<1$

$C_{6}=0\leq\mu^{a}<1$,$C_{7}=0\leq\mu^{a}<1$.

Furthermore consider the following recursive form given by:

$$X_{1n}\left(t\right)=S_{n}\left(t\right)-S_{n-1}\left(t\right)\frac{2\left(1-a\right)}{\left(2-a\right)Y\left(a\right)}\left[U_{1}\left(t,S_{n-1}\right)-U_{1}\left(t,S_{n-2}\right)\right]+\frac{2a}{\left(2-a\right)Y\left(a\right)}\int_{0}^{t} U_{1}\left(a,S_{n-1}\right)da,$$

$$X_{2n}\left(t\right)=E_{n}\left(t\right)-E_{n-1}\left(t\right)\frac{2\left(1-a\right)}{\left(2-a\right)Y\left(a\right)}\left[U_{2}\left(t,E_{n-1}\right)-U_{2}\left(t,E_{-2}\right)\right]+\frac{2a}{\left(2-a\right)Y\left(a\right)}\int_{0}^{t} U_{2}\left(a,E_{n-1}\right)da$$

$X_{3n}\left(t\right)=I_{n}\left(t\right)-I_{n-1}\left(t\right)\frac{2\left(1-a\right)}{\left(2-a\right)Y\left(a\right)}\left[U_{3}\left(t,I_{n-1}\right)-U_{3}\left(t_{1},I_{n-2}\right)\right]+\frac{2a}{\left(2-a\right)Y\left(a\right)}\int_{0}^{t} U_{3}\left(\alpha,I_{n-1}\right)da$ (13)

$$X_{4n}\left(t\right)=T_{n}\left(t\right)-T_{n-1}\left(t\right)\frac{2\left(1-a\right)}{\left(2-a\right)Y\left(a\right)}\left[U_{4}\left(t,T_{n-1}\right)-U_{4}\left(t,T_{n-2}\right)\right]+\frac{2a}{\left(2-a\right)Y\left(a\right)}\int_{0}^{t} U_{4}\left(a,T_{n-1}\right)da$$

$$G_{5n}\left(t\right)=R_{n}\left(t\right)-R_{n-1}\left(t\right)\frac{2\left(1-a\right)}{\left(2-a\right)Y\left(a\right)}\left[U_{5}\left(t,R_{n-1}\right)-U_{5}\left(t,R_{n-2}\right)\right]+\frac{2a}{\left(2-a\right)Y\left(a\right)}\int_{0}^{t} U_{5}\left(\alpha,R_{n-1}\right)da$$

$$X_{6n}\left(t\right)=V_{n}\left(t\right)-V_{n-1}\left(t\right)\frac{2\left(1-a\right)}{\left(2-\alpha\right)Y\left(a\right)}\left[U_{6}\left(t,V_{n-1}\right)-U_{6}\left(t,V_{n-2}\right)\right]+\frac{2a}{\left(2-a\right)Y\left(a\right)}\int_{0}^{t} U_{6}\left(\alpha,V_{n-1}\right)d\alpha$$

$$X_{7n}\left(t\right)=L_{n}\left(t\right)-L_{n-1}\left(t\right)\frac{2\left(1-a\right)}{\left(2-a\right)Y\left(a\right)}\left[U_{7}\left(t,L_{n-1}\right)-U_{7}\left(t_{1},L_{n-2}\right)\right]+\frac{2a}{\left(2-a\right)Y\left(a\right)}\int_{0}^{t} U_{7}\left(a,L_{n-1}\right)da$$

Subject to the initial conditions;

$$S_{0}\left(t\right)=h_{1}, E_{0}\left(t\right)=h_{2},I_{0}\left(t\right)=h_{3},T_{0}\left(t\right)=h_{4}, R_{0}\left(t\right)=h_{5}, V_{0}\left(t\right)=n_{6}, L_{0}\left(t\right)=n_{7}.$$

Norm of the above system to obtain$\left\|X_{1n}\left(t\right)\right\|=\left\|S_{n}\left(t\right)-S_{n-1}\left(t\right)\right\|=\left\|\frac{2\left(1-a\right)}{\left(2-a\right)Y\left(a\right)}\left[U_{1}\left(t,S_{n-1}\right)-U_{1}\left(t_{1},S_{n-2}\right)\right]+\frac{2a}{\left(2-a\right)Y\left(a\right)}\int_{0}^{t} U_{1}\left(\alpha,S_{n-1}\right)-U_{1}\left(t_{1},S_{n-2}\right)da\right\|,$ $\Rightarrow\left\|X_{1n}\left(t\right)\right\|\leq\frac{2\left(1-a\right)}{\left(2-a\right)Y\left(a\right)}\left\|\left[U_{1}\left(t,S_{n-1}\right)-U_{1}\left(t_{1},S_{n-2}\right)\right]\right\|+\frac{2a}{\left(2-a\right)Y\left(a\right)}\left\|\int_{0}^{t} U_{1}\left(a,S_{n-1}\right)-U_{1}\left(t_{1},S_{n-2}\right)da\right\|$.

Following the Lipchitz condition;

$\left\|X_{1n}\left(t\right)\right\|\leq\frac{2\left(1-a\right)}{\left(2-a\right)Y\left(\alpha\right)}C_{1}\left\|X_{1\left(n-1\right)}\left(t\right)\right\|+\frac{2a}{\left(2-a\right)Y\left(a\right)}C_{1}\times\int_{0}^{t} \left\|X_{1\left(n-1\right)}\left(a\right)\right\|da$,

$\left\|X_{2n}\left(t\right)\right\|\leq\frac{2\left(1-a\right)}{\left(2-a\right)Y\left(a\right)}C_{2}\left\|X_{2\left(n-1\right)}\left(t\right)\right\|+\frac{2a}{\left(2-a\right)Y\left(a\right)}C_{2}\times\int_{0}^{t} \left\|X_{2\left(n-1\right)}\left(a\right)\right\|da$.

In general, $\left\|X_{in}\left(t\right)\right\|\leq\frac{2\left(1-a\right)}{\left(2-a\right)Y\left(a\right)}C_{i}\left\|X_{i\left(n-1\right)}\left(t\right)\right\|+\frac{2a}{\left(2-a\right)Y\left(a\right)}C_{i}\times\int_{0}^{t} \left\|X_{i\left(n-1\right)}\left(a\right)\right\|da,$

$\begin{matrix} S_{n}\left(t\right)=\sum_{n=1}^{j} G_{1n}\left(t\right), E_{n}\left(t\right)=\sum_{n=1}^{j} G_{2n}\left(t\right), I_{n}\left(t\right)=\sum_{n=1}^{j} G_{3n}\left(t\right), T_{n}\left(t\right)=\sum_{n=1}^{j} G_{4n}\left(t\right), R_{n}\left(t\right)=\sum_{n=1}^{j} G_{5n}\left(t\right), \\ V_{n}\left(t\right)=\sum_{n=1}^{j} G_{6n}\left(t\right), L_{n}\left(t\right)=\sum_{n=1}^{j} G_{7n}\left(t\right). \end{matrix}$(14)

Thus, the system exists with unique and continuous solution

**S4. Laplace–Adomian Decomposition Method**

LADM on model equation (8) and exploiting the previous definitions further**.**

$$\begin{matrix} L\left[{}^{CF}D_{t}^{a}S(t)\right]=L\left[\Omega^{a}-\chi^{a}S(t)I(t)-\vartheta^{a}V(t)-\left(\mu+\varepsilon\right)^{a}S\left(t\right)\right], \\ L\left[{}^{CF}D_{t}^{a}E(t)\right]=L\left[\chi^{a}S(t)I(t)-\left(\mu+\varphi\right)^{a}E\left(t\right)\right], \\ L\left[{}^{CF}D_{t}^{a}I(t)\right]=L\left[\varphi^{a}E(t)-\left(\mu+\theta+\phi\right)^{a}I\left(t\right)\right], \\ L\left[{}^{CF}D_{t}^{a}T(t)\right]=L\left[\phi^{a}I(t)-\left(\mu+\tau\right)^{a}T\left(t\right)\right], (16) \\ L\left[{}^{CF}D_{t}^{a}R(t)\right]=L\left[\theta^{a}I(t)+\tau^{a}T\left(t\right)-\mu^{a}R(t)\right], \\ L\left[{}^{CF}D_{t}^{a}V(t)\right]=L\left[\theta^{a}(1-\beta)^{a}S(t)-(\mu+\vartheta)^{a}V(t)\right], \\ L\left[{}^{CF}D_{t}^{a}L(t)\right]=L\left[\theta^{a}\beta^{a}S(t)-\mu^{a}L(t)\right]. \end{matrix}$$

By definition, $L\left[_{0}^{CF}D^{\alpha}g\left(t\right)\right]=\frac{wL\left(g(t)-g(0)\right)}{w+a\left(1-w\right)}$for $0<w\leq1$.

Therefore,

$$\begin{matrix} \frac{wL\left(S(t)-S(0)\right)}{w+a\left(1-w\right)}=L\left[\Omega-\chi S(t)I(t)-\vartheta V(t)-\left(\mu+\varepsilon\right)S\left(t\right)\right], \\ \frac{wL\left(E(t)-E(0)\right)}{w+a\left(1-w\right)}=L\left[\chi S(t)I(t)-\left(\mu+\varphi\right)E\left(t\right)\right], \\ \frac{wL\left(I(t)-I(0)\right)}{w+a\left(1-w\right)}=L\left[\varphi E(t)-\left(\mu+\theta+\phi\right)I\left(t\right)\right], \\ \frac{wL\left(T(t)-T(0)\right)}{w+a\left(1-w\right)}=L\left[\phi I(t)-\left(\mu+\tau\right)T\left(t\right)\right], (17) \end{matrix}$$

$$\frac{wL\left(R(t)-R(0)\right)}{w+a\left(1-w\right)}=L\left[\theta I(t)+\tau T\left(t\right)-\mu R(t)\right],$$

$$\begin{matrix} \frac{wL\left(V(t)-V(0)\right)}{w+a\left(1-w\right)}=L\left[\theta(1-\beta)S(t)-(\mu+\vartheta)V(t)\right], \\ \frac{wL\left(L(t)-L(0)\right)}{w+a\left(1-w\right)}=L\left[\theta\beta S(t)-\mu L(t)\right]. \end{matrix}$$

Simplifying (17) with inverse Laplace transform of both sides yields:

$$\begin{matrix} S(t)=L^{-1}\left(\frac{S\left(0\right)}{w}\right)+L^{-1}\left(\frac{w+a\left(1-w\right)}{w}L\left[\Omega-\chi S(t)I(t)-\vartheta V(t)-\left(\mu+\varepsilon\right)S\left(t\right)\right]\right) \\ E(t)=L^{-1}\left(\frac{E\left(0\right)}{w}\right)+L^{-1}\left(\frac{w+a\left(1-w\right)}{w}L\left[\chi S(t)I(t)-\left(\mu+\varphi\right)E\left(t\right)\right]\right) \\ I(t)=L^{-1}\left(\frac{I\left(0\right)}{w}\right)+L^{-1}\left(\frac{w+a\left(1-w\right)}{w}L\left[\varphi E(t)-\left(\mu+\theta+\phi\right)I\left(t\right)\right]\right) \\ T(t)=L^{-1}\left(\frac{T\left(0\right)}{w}\right)+L^{-1}\left(\frac{w+a\left(1-w\right)}{w}L\left[\phi I(t)-\left(\mu+\tau\right)T\left(t\right)\right]\right) (18) \\ R(t)=L^{-1}\left(\frac{R\left(0\right)}{w}\right)+L^{-1}\left(\frac{w+a\left(1-w\right)}{w}L\left[\theta I(t)+\tau T\left(t\right)-\mu R(t)\right]\right) \\ V(t)=L^{-1}\left(\frac{V\left(0\right)}{w}\right)+L^{-1}\left(\frac{w+a\left(1-w\right)}{w}L\left[\theta(1-\beta)S(t)-(\mu+\vartheta)V(t)\right]\right) \\ L(t)=L^{-1}\left(\frac{L\left(0\right)}{w}\right)+L^{-1}\left(\frac{w+a\left(1-w\right)}{w}L\left[\theta\beta S(t)-\mu L(t)\right]\right) \end{matrix}$$

Assume that $S\left(t\right), E\left(t\right), I\left(t\right),T\left(t\right),R\left(t\right), V\left(t\right), L\left(t\right).$as an infinite series:

$\begin{matrix} S\left(t\right)=\sum_{n=0}^{\infty} S_{n}\left(t\right), E\left(t\right)=\sum_{n=0}^{\infty} E_{n}\left(t\right),I\left(t\right)=\sum_{n=0}^{\infty} I_{n}\left(t\right),T\left(t\right)=\sum_{n=0}^{\infty} T_{n}\left(t\right) \\ R\left(t\right)=\sum_{n=0}^{\infty} R_{n}\left(t\right), V\left(t\right)=\sum_{n=0}^{\infty} V_{n}\left(t\right), L\left(t\right)=\sum_{n=0}^{\infty} L_{n}\left(t\right). \end{matrix}$ (19)

Nonlinear terms are represented by:

$\sum_{n=0}^{\infty} Z_{n}\left(t\right)=S\left(t\right)I\left(t\right),$ (20)

Nonlinear terms are decomposed following Adomian polynomial by:

$Z_{n}\left(t\right)=\frac{1}{\Gamma\left(n+1\right)}\frac{d^{n}}{d\chi^{n}}\left[\sum_{j=0}^{n} B^{j}S_{j}\left(t\right)\sum_{j=0}^{n} \chi^{j}I_{j}\left(t\right)\right]$, (21)

The terms polynomials of $Z_{n}\left(t\right)$:

$$\begin{matrix} Z_{0}\left(t\right)=S_{0}(t)I_{0}(t), \\ Z_{1}\left(t\right)=S_{0}\left(t\right)I_{1}\left(t\right)+S_{1}\left(t\right)I_{1}\left(t\right), (22) \\ Z_{2}\left(t\right)=S_{0}\left(t\right)I_{2}\left(t\right)+S_{1}\left(t\right)I_{1}\left(t\right)+S_{2}\left(t\right)I_{0}\left(t\right) \end{matrix}$$

Evaluating (25) with subsequently simplifying by using the following $S\left(0\right)=h_{1}$,$E_{1}\left(0\right)=h_{2},$ $I\left(0\right)=h_{3},$ $T\left(0\right)=h_{4},$ $R\left(0\right)=h_{5},V\left(0\right)=h_{6},$ $L\left(0\right)=h_{7}.$ yields:

$$\begin{matrix} \sum_{n=0}^{\infty} S_{n+1}(t)=h_{1}+L^{-1}\left(\frac{w+a\left(1-w\right)}{w}L\left[\Omega-\chi\sum_{n=0}^{\infty} Z_{n}-\vartheta\sum_{n=0}^{\infty} V_{n}-\left(\mu+\varepsilon\right)\sum_{n=0}^{\infty} S_{n})\right]\right), \\ \sum_{n=0}^{\infty} E_{n+1}(t)=h_{2}+L^{-1}\left(\frac{w+a\left(1-w\right)}{w}L\left[\chi^{a}\sum_{n=0}^{\infty} Z_{n}\left(t\right)-\left(\mu+\varphi\right)^{\alpha}\sum_{n=0}^{\infty} E_{n}\left(t\right)\right]\right), \\ \sum_{n=0}^{\infty} I_{n+1}(t)=h_{3}+L^{-1}\left(\frac{w+a\left(1-w\right)}{w}L\left[\varphi^{a}\sum_{n=0}^{\infty} E_{n}\left(t\right)-\left(\mu+\theta+\phi\right)^{\alpha}\sum_{n=0}^{\infty} I_{n}\left(t\right)\right]\right), \\ \sum_{n=0}^{\infty} T_{n+1}(t)=h_{4}+L^{-1}\left(\frac{w+a\left(1-w\right)}{w}L\left[\phi^{a}\sum_{n=0}^{\infty} I_{n}\left(t\right)-\left(\mu+\tau\right)^{\alpha}\sum_{n=0}^{\infty} T_{n}\left(t\right)\right]\right), (23) \\ \sum_{n=0}^{\infty} R_{n+1}(t)=h_{5}+L^{-1}\left(\frac{w+a\left(1-w\right)}{w}L\left[\theta\sum_{n=0}^{\infty} I_{n}\left(t\right)+\tau^{\alpha}\sum_{n=0}^{\infty} T_{n}\left(t\right)-\mu^{\alpha}\sum_{n=0}^{\infty} R_{n}\left(t\right)\right]\right), \\ \sum_{n=0}^{\infty} V_{n+1}(t)=h_{6}+L^{-1}\left(\frac{w+a\left(1-w\right)}{w}L\left[\theta(1-\beta)\sum_{n=0}^{\infty} S_{n}\left(t\right)-(\mu+\vartheta)\sum_{n=0}^{\infty} V_{n}\left(t\right)\right]\right), \\ \sum_{n=0}^{\infty} L_{n+1}(t)=h_{7}+L^{-1}\left(\frac{w+a\left(1-w\right)}{w}L\left[\theta\beta\sum_{n=0}^{\infty} S_{n}\left(t\right)-\mu\sum_{n=0}^{\infty} L_{n}\left(t\right)\right]\right) \end{matrix}$$

Approximations

$S\left(0\right)=h_{1}$,$E_{1}\left(0\right)=h_{2},$ $I\left(0\right)=h_{3},$ $T\left(0\right)=h_{4},$ $R\left(0\right)=h_{5},V\left(0\right)=h_{6},$ $L\left(0\right)=h_{7}.$ Implementing these initial values to (26) at n=0 yields:

$$\begin{matrix} \sum_{n=0}^{\infty} S_{n+1}(t)=h_{1}+L^{-1}\left(\frac{w+a\left(1-w\right)}{w}L\left[\Omega-\chi\sum_{n=0}^{\infty} Z_{n}-\vartheta\sum_{n=0}^{\infty} V_{n}-\left(\mu+\varepsilon\right)\sum_{n=0}^{\infty} S_{n})\right]\right), \\ \sum_{n=0}^{\infty} E_{n+1}(t)=h_{2}+L^{-1}\left(\frac{w+a\left(1-w\right)}{w}L\left[\chi^{a}\sum_{n=0}^{\infty} Z_{n}\left(t\right)-\left(\mu+\varphi\right)^{\alpha}\sum_{n=0}^{\infty} E_{n}\left(t\right)\right]\right), \\ \sum_{n=0}^{\infty} I_{n+1}(t)=h_{3}+L^{-1}\left(\frac{w+a\left(1-w\right)}{w}L\left[\varphi^{a}\sum_{n=0}^{\infty} E_{n}\left(t\right)-\left(\mu+\theta+\phi\right)^{\alpha}\sum_{n=0}^{\infty} I_{n}\left(t\right)\right]\right), \end{matrix}$$

$$\begin{matrix} T_{1}(t)=L^{-1}\left(\frac{w+a\left(1-w\right)}{w}L\left[\phi^{a}\sum_{n=0}^{\infty} I_{n}\left(t\right)-\left(\mu+\tau\right)^{\alpha}\sum_{n=0}^{\infty} T_{n}\left(t\right)\right]\right), (24) \\ R_{1}(t)=L^{-1}\left(\frac{w+a\left(1-w\right)}{w}L\left[\theta\sum_{n=0}^{\infty} I_{n}\left(t\right)+\tau^{\alpha}\sum_{n=0}^{\infty} T_{n}\left(t\right)-\mu^{\alpha}\sum_{n=0}^{\infty} R_{n}\left(t\right)\right]\right), \\ V_{1}(t)=L^{-1}\left(\frac{w+a\left(1-w\right)}{w}L\left[\theta(1-\beta)\sum_{n=0}^{\infty} S_{n}\left(t\right)-(\mu+\vartheta)\sum_{n=0}^{\infty} V_{n}\left(t\right)\right]\right), \\ L_{1}(t)=L^{-1}\left(\frac{w+a\left(1-w\right)}{w}L\left[\theta\beta\sum_{n=0}^{\infty} S_{n}\left(t\right)-\mu\sum_{n=0}^{\infty} L_{n}\left(t\right)\right]\right) \end{matrix}$$

First approximate results are obtained as:

$$\begin{matrix} S_{1}\left(t\right)=\left(a\left(t-1\right)+1\right)\left(\Omega^{a}+\chi^{a}h_{1}h_{3}-h_{7}\left(\mu^{a}+\varepsilon^{a}\right)\right), \\ E_{1}\left(t\right)=\left(a\left(t-1\right)+1\right)\left(\chi^{a}hh_{1}h_{3}-h_{2}\left(\mu^{a}+\varphi^{a}\right)\right), \\ I_{1}\left(t\right)=\left(a\left(t-1\right)+1\right)\left(\varphi^{a}h_{2}-h_{3}\left(\mu^{a}+\theta^{a}+\phi^{a}\right)\right), (25) \\ T_{1}\left(t\right)=\left(a\left(t-1\right)+1\right)\left(\phi^{a}h_{3}-h_{4}\left(\mu^{a}+\tau^{a}\right)\right), \\ R_{1}\left(t\right)=\left(a\left(t-1\right)+1\right)\left(\theta^{a}h_{3}+\tau^{a}h_{4}-\mu^{a}h_{5}\right), \\ V_{1}\left(t\right)=\left(a\left(t-1\right)+1\right)\left(\theta(1-\beta)^{a}h_{1}-\mu^{a}h_{6}\right), \\ L_{1}\left(t\right)=\left(a\left(t-1\right)+1\right)\left(\theta\beta^{a}h_{1}-\mu^{a}h_{7}\right). \end{matrix}$$

The further process is done by repeating this after which the approximate results of the model are calculated.

$\begin{matrix} \left(t\right)=\sum_{n=0}^{3} S_{n}\left(t\right), E\left(t\right)=\sum_{n=0}^{3} E_{n}\left(t\right), I\left(t\right)=\sum_{n=0}^{3} I_{n}\left(t\right), T\left(t\right)=\sum_{n=0}^{3} T_{n}\left(t\right), R\left(t\right)=\sum_{n=0}^{3} R_{n}\left(t\right), \\ V\left(t\right)=\sum_{n=0}^{3} V_{n}\left(t\right), L\left(t\right)=\sum_{n=0}^{3} L_{n}\left(t\right) \end{matrix}$(26)

**S5. Convergence Analysis**

**Convergence Analysis**

The Laplace -Adomian series in equation (26) has a rapid and consistent convergence to the exact solution of the system. In order to illustrate this we prove the convergence of the solution by the method of [39].

Theorem **S3.2** [39] (Contraction and convergence of the LADM iteration)

Let $\Pi$ be the Banach Space of continuous vector –valued function on (0.T] equipped with the supremum norm [29]. Consider the Caputo–Fabrizio system written in the equivalent integral form along with $u(t)=u_{0}+\frac{2(1-\Pi)}{(2-\Pi)N(\Pi)}\int_{0}^{t} F(s,u(s))ds, t\in(0,T)$

Where $u(t)=R^{n}$the state is vector and $f(t)$ contains the nonlinear coupling term of the model.

Define the LADM iteration operator $X:\Pi\to\Pi$ by

$$(Xu)(t)=u_{0}+\frac{2(1-\Pi)}{(2-\Pi)N(\Pi)}\int_{0}^{t} F(s,u(s))ds, t\in(0,T)$$

Assume $G$is Lipschitz in$u$: there exists a constant $L>0$ such that $\left\|G(t,u)-G(t,v)\right\|\leq L\left\|u-v)\right\|, \forall t \in(0,T), u,v\in R^{n}.$

Note that a similar assumption is made in [43], Let $\delta=\frac{2(1-\Pi)}{(2-\Pi)N(\Pi)}LT$

If $\delta<1$ then $X$ is a strict contraction on $\Pi$ and therefore has a unique fixed point $u$in$\Pi$. In particular, the LADM iteration (Picard iteration)

$u_{0}(t)=u_{0} u_{k+1}(t)=X(u_{k})(t).$ Generates a sequence that converges in $\Pi$ to the unique solution $u(t)$ of the integral equation (and hence of the original CF model).

Proof. For any $u,v$ in $\Pi$ and $t in [0.T]$ the Lipschitz assumption and the definition of $X$give

$$\left\|X(u)t-X(v)t\right\|=\frac{2(1-\Pi)}{(2-\Pi)N(\Pi)}\left\|\int_{0}^{t} \left[G(s,u(s))-G(s,v(s))\right]ds\right\|.$$

Using the sup-norm on $\Pi$ and the Lipschitz bound, we estimate

$$\left\|X(u)t-X(v)t\right\|\leq\frac{2(1-\Pi)}{(2-\Pi)N(\Pi)}\left\|\int_{0}^{T} \left[G(s,u(s))-G(s,v(s))\right]ds\right\|\leq\frac{2(1-\Pi)}{(2-\Pi)N(\Pi)}LT\left\|u-v\right\|$$

By definition, $\frac{2(1-\Pi)}{(2-\Pi)N(\Pi)}LT$. Under the hypothesis $\delta<1$, this shows

$$\left\|X(u)t-X(v)t\right\|\leq\delta\left\|u-v\right\| u,v\in\Pi$$

so $X$ is a contraction mapping on the complete space $\Pi$. Hence by the Banach fixed-point theorem, $X$ has a unique fixed point $u$in $\Pi$ satisfying $X_{u}=u^{*}$ This fixed point is exactly the (unique) solution of the integral equation and thus of the original CF system.

Moreover, the iterative scheme $u_{k+1}=u_{k}$ satisfies

$\left\|u_{k+1}-u_{k}\right\|\leq\delta\left\|u_{k+1}-u_{k}\right\| \leq\delta^{k}\left\|u_{1}-u_{0}\right\|$.

Because$\delta<1$, the series of partial sums $u_{k}$is Cauchy and converges to $u^{*}$at a geometric rate. In other words, the LADM series solution converges in norm to the unique solution of the model. This completes the proof of Theorem 2

Although the convergence proof uses the Banach fixed-point theorem, it is not redundant because it adapts to the Caputo–Fabrizio operator’s non-singular kernel, handles nonlinear multi-compartment coupling in the model-specific operator $\Pi$, and establishes a concrete convergence condition $\delta<1$ linking parameters like the CF order and time T. This ensures that the Laplace–Adomian method remains valid and stable for this specific corruption model and parameter regime**.**
